# Recent Advances in Supramolecular Chemistry

**DOI:** 10.3390/molecules31101556

**Published:** 2026-05-07

**Authors:** Wen-Chao Geng

**Affiliations:** State Key Laboratory of Green Biomanufacturing, College of Life Science and Technology, Beijing University of Chemical Technology, Beijing 100029, China; wchgeng@buct.edu.cn

## 1. Introduction

Supramolecular chemistry, founded on the non-covalent interactions between molecular building blocks, has evolved into one of the most vibrant and interdisciplinary fields of modern chemistry [1,2]. The profound impact of supramolecular chemistry has been recognized twice by the Nobel Prize in Chemistry: first in 1987, when Cram, Lehn, and Pedersen were awarded for their pioneering work on host–guest chemistry [3], and again in 2016 for the design and synthesis of molecular machines [4]. At the heart of this discipline lie macrocyclic hosts with versatile molecular platforms with preorganized cavities, tunable chemical functionalities, and unique recognition properties [5,6,7]. From the classic crown ethers, cyclodextrins, and calixarenes to emerging pillararenes, cucurbiturils, and resorcinarene derivatives, macrocyclic compounds have continuously pushed the boundaries of supramolecular chemistry, enabling breakthroughs in applications including adsorption, ion transportation, biomedicine, and catalysis [8,9,10,11].

This Special Issue, entitled *Recent Advances in Supramolecular Chemistry*, compiles six original research articles and one review that showcase the latest advances in the field. The contributions span from the rational design of novel macrocyclic receptors and mechanistic elucidation of supramolecular assembly processes to the development of functional macrocycle-based materials. Collectively, these works highlight the inherent versatility of macrocyclic systems, bridging fundamental supramolecular chemistry with real-world applications and integrating synthetic organic chemistry, computational simulation, materials science, and biomedical research.

## 2. Advances in Macrocyclic Receptor Design and Molecular Recognition

Molecular recognition, the cornerstone of supramolecular chemistry, relies on the precise spatial matching and non-covalent interaction complementarity between host and guest molecules. A contribution in this Special Issue focuses on the design of novel macrocyclic receptors with tailored recognition properties for targeted guest species.

Maldonado et al. (Contribution 1) report the synthesis, characterization, and thermodynamic properties of cyclocondensation products of pyrogallol and propanaldehyde. The authors isolate and purify two main products: the crown conformer of C-tetra(ethyl)pyrogallol[4]arene and its dimeric hydrogen-bonded capsule, which are fully characterized by NMR spectroscopy, mass spectrometry, and FT-IR. Volumetric property studies in dimethyl sulfoxide solution reveal drastic differences in the apparent molar volume and standard molar expansibility between the monomeric crown conformer and the dimeric capsule, providing quantitative insights into solute–solvent interactions and structural effects of macrocyclic assembly. This work offers the first thermodynamic characterization of a pyrogallolarene dimeric capsule, shedding light on the relationship between supramolecular structure and solution thermodynamic behavior.

## 3. Mechanistic Elucidation of Supramolecular Complexation

Understanding the dynamic self-assembly processes of macrocyclic compounds and the structure–property relationships of the resulting assemblies is critical for the rational design of functional supramolecular systems. Two contributions in this Special Issue combine computational simulation and experimental characterization to unravel the assembly mechanisms and thermodynamic properties of resorcinarene- and pyrogallolarene-based supramolecular architectures.

Schurhammer and Sémeril present a comprehensive review of in silico techniques for the study of confined supramolecular systems based on resorcin[4]arenes, pyrogallol[4]arenes, velcrands, and octa acid assemblies (Contribution 2). The work highlights how computational methods, including molecular dynamics, metadynamics, density functional theory (DFT), and ONIOM hybrid calculations, provide atomic-level insights into capsule formation, dynamic behavior, guest encapsulation, and catalytic reaction mechanisms within these confined cavities, often revealing details inaccessible to experimental techniques. The authors systematically discuss the ability of in silico studies to map the early-stage assembly intermediates of hexameric resorcinarene capsules, quantify the role of structural water molecules in assembly stability, and explain the origin of catalytic selectivity in confined nanospaces. This review underscores the indispensable role of computational chemistry in modern supramolecular research, enabling predictive design of supramolecular assemblies prior to experimental validation.

Kinart, Hoelm, and Imińska conducted a systematic evaluation of four widely used implicit solvent models, including PCM, CPCM, SMD, and Onsager, for dacarbazine (DTIC)–cyclodextrin inclusion complexes, combining DFT calculations with conductometric experiments (Contribution 3). DTIC is an FDA-approved anticancer drug with clinical limitations from poor water solubility, and cyclodextrin complexation is the key strategy used to address this issue. The authors found that solvent models have negligible impact on complex geometry but profoundly affect thermodynamic parameters; the SMD model showed the best agreement with experimental data, especially for solvation free energy. They also confirmed that all three tested cyclodextrins form stable 1:1 inclusion complexes with DTIC, with HE-β-CD forming the most thermodynamically stable complexes. This work establishes a validated computational benchmark for the in silico design of cyclodextrin-based drug delivery systems.

## 4. Supramolecular Materials for Functional Applications

The ultimate goal of supramolecular chemistry is to translate molecular recognition and assembly into functional materials with practical utility. The remaining four contributions in this Special Issue explore the application of supramolecular materials in four important fields, including industrial adsorptive separation, ion transportation, biomedicine, and cancer photodynamic therapy.

### 4.1. Adsorptive Separation

Wu et al. (Contribution 4) developed nonporous adaptive crystals (NACs) of perethylated pillar[6]arene (EtP6) for the highly efficient separation of chlorobenzene (CB) and chlorocyclohexane (CCH), which is a major industrial challenge due to their close boiling points. The authors demonstrate that EtP6 NACs can selectively adsorb CCH from an equimolar vapor mixture of CB and CCH, with a purity of up to 99.5%. Single-crystal X-ray diffraction and DFT calculations reveal that the exceptional selectivity originates from the stronger host–guest interactions and higher thermodynamic stability of the CCH@EtP6 complex compared to the CB-loaded counterpart. Notably, the material can be recycled for at least five cycles without significant loss of separation performance, offering a low-energy, sustainable alternative to energy-intensive distillation for industrial aromatic/alicyclic separation. This work expands the application scope of pillararene-based NACs and provides a new strategy for the design of supramolecular adsorbents for challenging industrial separations.

### 4.2. Ion Transportation

The contribution from Torres-Huerta and Valkenier addresses a long-standing gap in our understanding of organic anion transport across lipid membranes, a process central to cellular metabolism, drug absorption, and the treatment of ion channelopathies (Contribution 5). Carboxylates are ubiquitous in biological systems, forming the core structure of amino acids, Krebs cycle metabolites, and over 25% of marketed small-molecule drugs. However, the structural diversity of carboxylates has hindered a comprehensive understanding of how their organic moieties modulate transmembrane diffusion and transporter-assisted transport, with prior studies limited to a narrow range of carboxylate structures. In this work, using fluorescence assays and chloride-ion-selective electrode measurements, the authors decoupled the two transport processes and proposed a three-step diffusion mechanism for carboxylates in neutral nitrate media. Key findings include that hydroxyl substitution significantly enhances carboxylate transport efficiency, with the position of hydroxyl groups rather than their number dominating transport performance. This work establishes a standardized framework for organic anion transport research and provides design principles for improving the membrane permeability of carboxylate-based drugs.

### 4.3. Biomedicine

Song et al. (Contribution 6) construct a novel supramolecular photosensitizer based on partially methyl-substituted cucurbit[6]uril (HMeQ[6]) and a porphyrin derivative (DPPY) for the photodynamic therapy of triple-negative breast cancer (TNBC). The authors show that HMeQ[6] and DPPY self-assemble into a 2:1 supramolecular complex via host–guest interactions, with a binding constant of 2.11 × 10^5^ M^−1^. Encapsulation by HMeQ[6] effectively inhibits the π–π stacking aggregation of DPPY, resulting in a three-fold enhancement in fluorescence intensity and a six-fold increase in singlet oxygen generation efficiency compared to free DPPY. In vitro studies using 4T1 TNBC cells demonstrate efficient cellular uptake of the complex, which induces concentration-dependent reactive oxygen species production and apoptosis upon light irradiation, with a half-maximal inhibitory concentration of 1.8 μM and minimal dark toxicity. This work provides a straightforward supramolecular strategy to overcome the aggregation-caused quenching effect of porphyrin photosensitizers and opens new avenues for the development of cucurbituril-based supramolecular theranostic systems.

### 4.4. Asymmetric Catalysis

Guo et al. (Contribution 7) developed a straightforward strategy to construct DNA-like double-helical supramolecular chiral polymers, using tyrosine-functionalized pillar[5]arene as a chiral inducer, a chiral quinoline monomer, and Cu(I) as the coordination center. Only 7 mol% of the chiral inducer enabled the formation of enantiomerically enriched (M)- and (P)-Helix systems with excellent chiroptical stability and significant chirality amplification. In the intermolecular cyano-trifluoromethylation of olefins, the (M)-Helix catalyst achieved an isolated yield of up to 89%, while the (P)-Helix delivered an enantiomeric excess of up to 84%. This work avoids the tedious synthesis of complex chiral ligands, opening a new avenue for pillararene-based supramolecular systems in industrial asymmetric catalysis.

## 5. Summary and Outlook

The seven articles collected in this Special Issue exemplify the remarkable diversity and vitality of contemporary macrocyclic supramolecular chemistry. From the rational design of new macrocyclic receptors for molecular recognition and sensing, to the mechanistic elucidation of supramolecular assembly via computational and experimental approaches, to the development of functional supramolecular materials, these contributions cover the full spectrum of modern supramolecular research. They highlight a key trend of the increasing integration of fundamental supramolecular chemistry with cross-disciplinary fields, including computational chemistry, environmental science, materials science, and biomedicine.

While significant progress has been made, many challenges and opportunities remain. The development of novel macrocyclic hosts with unique recognition properties, the construction of intelligent supramolecular systems with dynamic and adaptive behavior, and the translation of lab-scale supramolecular materials into real-world industrial and clinical applications will continue to be the focus of future research. We hope that this Special Issue will not only showcase the latest advances in macrocyclic supramolecular chemistry but also inspire new ideas and collaborations in this exciting and ever-evolving field.
